# Perioperative discordance in mesothelioma cell type after pleurectomy/decortication—a possible detrimental effect of neoadjuvant chemotherapy due to epithelial to mesenchymal transition?

**DOI:** 10.1093/icvts/ivad145

**Published:** 2023-10-18

**Authors:** Luigi Ventura, Michelle Lee, Ralitsa Baranowski, Joanne Hargrave, Michael Sheaff, David Waller

**Affiliations:** Barts Thorax Centre, St Bartholomew's Hospital, London, UK; Barts Thorax Centre, St Bartholomew's Hospital, London, UK; Barts Thorax Centre, St Bartholomew's Hospital, London, UK; Barts Thorax Centre, St Bartholomew's Hospital, London, UK; Barts Thorax Centre, St Bartholomew's Hospital, London, UK; Barts Thorax Centre, St Bartholomew's Hospital, London, UK

**Keywords:** Mesothelioma, Pleurectomy/decortication, Epithelial-to-mesenchymal transition

## Abstract

**OBJECTIVES:**

The goal was to evaluate the accuracy of preoperative histological assessment and the factors affecting the accuracy and the subsequent effect on postoperative survival after surgical treatment for malignant pleural mesothelioma (MPM).

**METHODS:**

We analysed the perioperative course of patients who underwent surgery for MPM in a single institution over a 5-year period. The primary end point was to evaluate the proportion of histological discordance between preoperative assessment and postoperative histological diagnosis. The secondary end point was to evaluate its prognostic effect on postoperative survival after surgical treatment.

**RESULTS:**

One-hundred and twenty-nine patients were included in this study. Histological discordance between preoperative assessment and postoperative histological diagnosis was found in 27 of 129 patients (20.9%): epithelial to biphasic/sarcomatoid (*negative discordance*) in 24 and biphasic to epithelial (*positive discordance*) in 3 (*P*-value < 0.001). All 24 patients who exhibited epithelial-to-mesenchymal transition (EMT) had received neoadjuvant chemotherapy (*P*-value: 0.006). In the 34 patients who underwent upfront surgery, only 1 case (2.9%) of EMT was identified (*P*-value: 0.127). EMT was not associated with a less invasive method of biopsy (*P*-value: 0.058) or with the volume or maximum diameter of the biopsy (*P*-value: 0.358 and 0.518, respectively), but it was significantly associated with the receipt of neoadjuvant chemotherapy (*P*-value: 0.006). At a median follow-up of 17 months (IQR: 11.0–28.0), 50 (39%) patients are still alive. Overall survival was significantly reduced in those patients who received neoadjuvant chemotherapy and who exhibited discordance (EMT) compared to those who did not: 11 (95% CI: 6.2–15.8) months versus 19 (95% CI: 14.2–23.8) months (*P*-value < 0.001). In addition, there was no difference in overall survival between those who received neoadjuvant chemotherapy and those who had upfront surgery: 16 (95% CI: 2.5–19.5) months versus 30 (95% CI: 11.6–48.4) months (*P*-value: 0.203).

**CONCLUSIONS:**

The association of neoadjuvant chemotherapy with perioperative histological discordance can be explained by EMT, which leads to worse survival. Therefore, there is an argument for the preferential use of upfront surgery in the treatment of otherwise resectable MPM.

**Figure:**
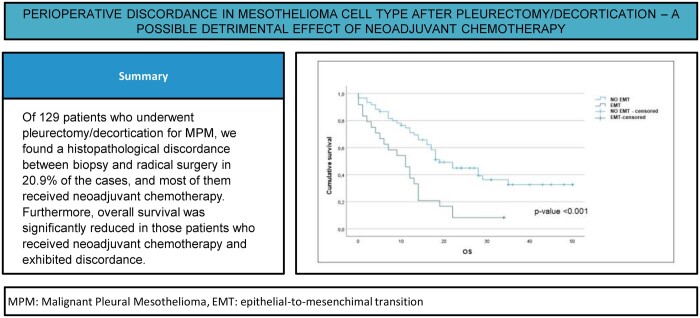

## INTRODUCTION

Treatment decisions regarding radical surgery in patients with mesothelioma are based on preoperative assessment of prognostic variables. A non-epithelial cell type is associated with poorer prognosis [1]; surgery in patients with pure sarcomatoid disease does not influence survival [2]; and, for this reason, surgery is not generally recommended. In patients with biphasic mesothelioma there remains much debate regarding the use of surgery [3]. A preoperative diagnosis is made using a variety of both invasive or non-invasive approaches ranging from a percutaneous image guided by ultrasound (US) or computed tomography (CT) to thoracoscopy with the patient under local or general anaesthesia. Histological discordance between tumour cell type diagnosed on a preoperative biopsy and post-resectional histological diagnosis has been demonstrated previously [4, 5]. This finding has focused most attention on the process and effectiveness of biopsy techniques [6]. Our goal was to evaluate the comparative accuracy of contemporary methods of preoperative histological assessment and to examine how a wider range of factors may contribute to histological discordance. We also evaluated the subsequent effect on postoperative survival specifically after pleurectomy/decortication (PD) or extended pleurectomy/decortication (EPD) and on identifying possible improvements in the preoperative workup that could prolong survival.

## METHODS

We analysed the perioperative course of patients who underwent radical surgery for malignant pleural mesothelioma (MPM) by a single surgeon in a single institution over a 5-year period (from January 2017 to December 2021). External referring centres were also involved, with different pathology centres responsible for reporting the pathological results of the biopsy samples.

### Diagnostic procedure

All patients had preoperative biopsy-confirmed mesothelioma as reported by the mesothelioma specialist pathologist in each referring centre. Tissue was obtained by an image-guided (CT or US) percutaneous biopsy method and by medical and surgical thoracoscopy.

### Surgical procedure

The objective of the operation was to achieve macroscopic complete resection with preservation of as much lung tissue and diaphragmatic muscle as possible [7]. Resectability was determined by the ability to achieve macroscopic complete resection, which included resectable nodal disease (N1). Non-epithelial disease was resected in the clinical absence of nodal disease. Invasive mediastinal staging was not routinely employed. Upfront surgery was offered in cT1/T2N0M0 patients with cT1/T2N0M0 disease (*surgery-first* subgroup), whereas in patients with more advanced tumour stages, a neodjuvant chemotherapy was administered (*first-line chemotherapy* subgroup).

### Additional chemotherapy

Surgery was offered as part of multimodality therapy including platinum/pemetrexed chemotherapy. The timing of additional chemotherapy was determined by local protocol, and the decision making was independent of the surgical team. Post-induction resectability was confirmed by restaging CT, but no further biopsy was performed.

### Histopathological classification

All the pathology slides (preoperative and postoperative) obtained during the study period were retrospectively reviewed by 2 pathologists from our thoracic oncology group and reclassified according to the *TNM Classification of Malignant Tumours, 8^th^ edition* (published by Wiley-Blackwell in affiliation with the Union for International Cancer Control; 2017). All cases were also pathologically staged according to the eighth edition of the International Union Against Cancer (UICC)/American Joint Committee on Cancer TNM classification. In particular, we recorded histological discordance between preoperative assessment and postoperative findings after resection. All the pathologists involved were consultants, and any tissue biopsy of insufficient size was reported. Based on the histopathology report, all the biopsies satisfied the basic requirements. Furthermore, the cases were initially reviewed by the 2 pathologists in a double-blind manner. There were a few discordant cases. However, Cohen's kappa coefficient was superior to 0.81 (almost perfect agreement). These cases were reviewed in consensus by the 2 pathologists, and a new agreed-on pattern was assigned. Patients were followed up with clinical and radiological reviews until death. Specifically, all the follow-up data were collected through NHS Spine (https://digital.nhs.uk/services/spine), which is a national digital database that links all NHS providers across the United Kingdom. Other follow-up data not available on NHS Spine were collected through the NHS Care Record Service (NCRS - https://digital.nhs.uk/services/national-care-records-service). Overall survival from date of diagnosis was recorded.

### Definition of the *concordant* and *discordant* subgroups

We defined the subgroup of patients who had the same histopathological pattern between the biopsy and the surgical procedure as *concordant*. In contrast, the *discordant* subgroup was composed of patients in whom the histopathological pattern from the biopsy differed from that from the surgical procedure. Within the *discordant* subgroup, a *positive discordance* was a non-epithelioid pattern on the biopsy and an epithelioid pattern diagnosed after the surgical procedure. A *negative* discordance was defined as the shift from a non-epithelioid pattern on the biopsy to an epithelioid pattern after the surgical procedure.

Based on the goal of the study, the *concordance* and the *discordance* subgroups were compared as were the *surgery-first* versus the *first-line chemotherapy* subgroups.

### Statistical analyses

Categorical variables are reported as frequencies and proportions. Continuous variables are reported as means [± standard deviation (SD)] and medians [± interquartile ranges (IQR)]. For comparing the baseline characteristics between the groups, we used the Student *t*-test for normally distributed variables, the χ^2^ test or the Fisher exact test for categorical variables and the Mann–Whitney U test for all other continuous variables. The Kaplan–Meier method was used to estimate the overall survival, and the log-rank test was used to compare the survival curves. A univariable and a multivariable Cox regression analysis with the step-wise method were performed to estimate the hazard ratios (HRs) and 95% confidence intervals (CIs) of each variable for the overall survival (OS) calculation. All statistical calculations were performed using SPSS 22.0 (SPSS, Chicago, IL, USA). All *P*-values were two-tailed, and values of less than 0.05 were considered statistically significant.

### Ethical statement

This study is registered as a clinical audit in the Department of Thoracic Surgery at Barts Health NHS Trust with an audit ID 13012. The Ministry of Ethics of the United Kingdom states that when a clinical audit does not alter patients’ usual clinical management, it does not require additional patient consent or formal ethical review or approval from the NHS Research Ethics Service [8]. However, according to the recommendations of the International Committee of Medical Journal Editors, the study has also been approved by the local ethics committee (protocol number: 2520).

## RESULTS

One hundred and twenty-nine patients [108 male, 21 female; median age: 68.0 (IQR: 63.0–73.0) years] who underwent radical surgery for MPM by a single surgeon in a single institution over a 5-year period were included in this study. Forty-nine referring centres were involved, with 46 different pathology centres responsible for reporting the pathological results of the biopsy samples. The clinicopathological characteristics of the entire population are shown in Table 1.

**Table 1:** Clinicopathological characteristics of the study population

Age, years (mean ± SD) (median–percentiles)	67.12 (SD: 7.9) 68.00 (63.00–73.00)
Gender	
Male	108 (83.7%)
Female	21 (6.3%)
Smoking history	
Never	49 (38%)
Former + current	80 (62%)
ECOG	
0	70 (54.3%)
1 + 2	59 (55.7%)
Pre-op Hb	
≤130	85 (65.9%)
>130	44 (34.1%)
Pre-op BMI	
<25	42 (32.5%)
≥25	87 (67.5%)
Biopsy method	
VATS	79 (61.2%)
Medical thoracoscopy	28 (21.7%)
Percutaneous CT/US guided	22 (17.1%)
Biopsy–histological diagnosis	
Epithelioid	117 (90.7%)
Other	12 (9.3%)
Induction chemotherapy	
No	34 (26.4%)
Yes	95 (73.6%)
Surgical procedure	
PD	32 (24.8%)
EPD	97 (85.2%)
Interval biopsy–surgery (months)	4.71±4.19
Surgery–histological analysis	
Epithelioid	96 (74.4%)
Non-epithelioid	33 (25.6%)
Stage 8TNM	
IA+IB	58 (45%)
II+III	71 (55%)
Surgery–Sarcomatoid component (%)	
≤20%	104 (80.6%)
>20%	25 (17.8%)
N status	
N0	63 (48.8%)
N+	66 (51.1%)

BMI: body mass index; CT/US: computed tomography/ultrasound; ECOG: Eastern Cooperative Oncology Group; EPD: extended pleurectomy/decortication; Hb: haemoglobin; N: node; PD: pleurectomy/decortication; pre-op: preoperative; SD: standard deviation; TNM: tumour/node/metastasis; VATS: video-assisted thoracoscopic surgery.

### Histological analysis

The preoperative histological analysis showed epithelial cell types in 90.7% and non-epithelial cell types in 9.3%. Post-resectional histological analysis showed epithelial cells in 74.5% and non-epithelial cells in 25.6%. In 27 of 129 (20.9%) patients, the histological subtype in the resection specimen did not correlate with that in the biopsy report. Three of 129 (2.3%) patients exhibited *positive discordance* from biphasic/sarcomatoid histological analysis of the preoperative biopsy to the epithelioid subtype after surgical resection, with a presumed better prognosis. In contrast, 24 of 129 (18.6%) patients had *negative discordance* from an epithelioid tumour that was biopsied to a biphasic result after resection or from biphasic on the biopsied tissue to pure sarcomatoid after resection (*P*-value < 0.001– Fisher exact test; Table 2). In the 31 patients with post-resection biphasic disease, the proportion of the sarcomatoid component was ≤20% in 9 patients and >20% in 22 patients.

**Table 2:** Evaluation of the *concordant* and *discordant* events passing from the biopsy to the surgical procedure in the entire study population (2-sided Fisher's exact test; *P*-value < 0.001).

Histology after surgery			
Histology after biopsy	Epithelioid	Biphasic+sarcomatoid	Total
Epithelioid	93	24	117
Non-epithelioid	3	9	12
Total	96	33	129

### Comparison between *concordant* and *discordant* subgroups

The 29 patients with histopathological discordance were older [68-96 years (SD: 9.452) vs 66–67 (SD: 7.351); *P*-value: 0.052] but had otherwise similar preoperative clinical features compared to the remaining patients in whom there was a histopathological concordance between the biopsy and the surgical procedure (Table 3).

**Table 3:** Clinicopathological characteristics classified by *concordant* versus *discordant* subgroups

	Concordance	Discordance	*P*-value
	(n = 102)	(n = 27)	
Age, years (mean+/-SD) (median–percentiles)	66.67 (SD: 7.35)	68.96 (SD: 9.45)	0.052
Gender			1.000
Male	85 (83.3%)	23 (85.2%)	
Female	17 (16.7%)	4 (14.8%)	
Smoking history			0.096
Never	35 (34.3%)	14 (51.9%)	
Former + current	67 (65.7%)	13 (48.1%)	
ECOG			0.827
0	54 (52.9%)	16( 59.2%)	
1 + 2	48 (47.1%)	11 (40.8%)	
Pre-op HB			0.116
≤130	64 (62.7%)	21 (77.8%)	
>130	38 (37.3%)	6 (22.2%)	
Pre-op BMI			0.638
<25	31 (30.4%)	11 (40.7%)	
≥25	71 (69.6%)	16 (59.3%)	
Biopsy method			0.058
VATS	62 (60.8%)	17 (63.0%)	
Medical thoracoscopy	19 (18.6%)	9 (33.3%)	
Percutaneous CT/US guided	21 (20.6%)	1 (3.7%)	
Biopsy volume (mean ± SD, mm^3^)	2750 (SD: 662)	2438 (SD: 730)	0.358
Biopsy size (mean ± SD, mm)	30.5 (SD: 21.1)	37.8 (SD: 29.4)	0.518
			
Biopsy:_Histological type			0.703
Epithelioid	93 (91.2%)	24 (88.9%)	
Non-epithelioid	9 (8.8%)	3 (11.1%)	
Surgical procedure			0.002
PD	31 (30.4%)	1 (3.7%)	
EPD	71 (69.6%)	26 (96.3%)	
Interval biopsy–surgery (months)	4.82+/-4.67	4.27+/-2.16	0.710
Surgery:_Histological type			<0.001
Epithelioid	92 (90%)	4 (11.1%)	
Non-epithelioid	10 (10%)	23 (88.9%)	
Sarcomatoid component			<0.001
≤20%	94 (92.2%)	10 (37%)	
>20%	8 (7.8%)	17 (63%)	
Stage 8TNM			0.192
IA + IB	49 (47.1%)	9 (33.3%)	
II + III	53 (52.9%)	18 (66.7%)	
N Status			0.384
N0	52 (51%)	11 (40.7%)	
N+	50 (49%)	16 (49.3%)	

BMI: body mass index; CT/US: computed tomography/ultrasound; ECOG: Eastern Cooperative Oncology Group; EPD: extended pleurectomy/decortication; Hb: haemoglobin; N: node; PD: pleurectomy/decortication; pre-op: preoperative; SD: standard deviation; TNM: tumour/node/metastasis; VATS: video-assisted thoracoscopic surgery.

### Biopsy method

All patients had preoperative biopsy-confirmed mesothelioma as reported by the mesothelioma specialist pathologist in each referring centre. Tissue was obtained by an image-guided (CT or US) percutaneous biopsy method in 22 cases and by thoracoscopy in 107 cases [79 video-assisted thoracic surgeries (VATS) vs 28 local anaesthetic thoracoscopy (LAT)].

Between these 2 subgroups of patients there were no significant differences in terms of biopsy methods even though VATS and percutaneous CT/US guided biopsies were the preferred methods in the concordant group, whereas VATS and medical thoracoscopy were used more frequently in the discordant group (*P*-value: 0.058). In the discordant group, 17 patients (63%) had a VATS biopsy; 9 (33.3%) had a biopsy via medical thoracoscopy;, and only 1 (3.7%) had a CT-guided percutaneous biopsy. In addition, there was no difference in the volume and size of the biopsy between the concordance and the discordance groups [biopsy volume: 2750 ± (SD: 662) vs 2438 (SD: 730) mm^3^, *P*-value: 0.358—biopsy size: 30.5 (SD: 21.1) vs 37.8 (SD: 29.4) mm, *P*-value: 0.518, respectively].

### Surgery, tumour stage

More patients in the discordant group were treated by EPD than in the concordant group (*P*-value: 0.002). Maximal tumour thickness on initial staging CT (prior to any therapy) was not associated with cell type discordance: concordant 13 (3–66) mm vs discordant 16 (5–35) mm, *P*-value: 0.135. Similarly, tumour stage and lymph node status were not associated with histopathological discordance (Table 3). As previously stated, post-resection non-epithelial disease was more frequent in the discordant group (88.9% vs 10%; *P*-value <0.001). Consequently, the sarcomatoid component was more prominent in this subgroup (63% vs 7.8%; *P*-value <0.001).

### Induction chemotherapy

Ninety-five patients had neoadjuvant chemotherapy [median number of cycles = 2 (IQR: 2–3)] whereas 34 had upfront surgery followed by adjuvant chemotherapy. Post-induction resectability was confirmed by restaging CT but no further biopsies were performed.

Discordance was significantly associated with the use of neoadjuvant chemotherapy. In those patients who underwent neoadjuvant chemotherapy, *negative discordance* was observed in 24 of 93 (25.8%), whereas only 2 of 93 (2.15%) demonstrated *positive discordance* (*P*-value: 0.006, Fisher exact test; Table 4). In contrast, in patients who underwent an upfront operation, in the interval between the diagnostic procedure and the operation, only 1 case (2.8%) of epithelial-to-mesenchymal transition (EMT) was reported (*P*-value: 0.127; Fisher exact test; Table 5).

**Table 4:** Evaluation of the *concordant* and *discordant* events passing from the biopsy to the surgical procedure in the *first-line chemotherapy* study population (two-sided Fisher's exact test; *P*-value: 0.006).

Histology after surgery			
Histology after biopsy	Epithelioid	Biphasic+sarcomatoid	Total
Epithelioid	61	24	85
Percent histological type after biopsy	2	8	10
TOTAL	63	32	95

**Table 5:** Evaluation of the *concordant* and *discordant* events passing from the biopsy to the surgical procedure in the *surgery-first* study population (two-sided Fisher's exact test; *P*-value: 0.127).

Histology after surgery			
Histology after biopsy	Epithelioid	Biphasic+sarcomatoid	Total*
Epithelioid	31	1	32
Percent histology after biopsy	1	1	2
Total	32	2	34

* Two-sided Fisher's exact test; *P*-value: 0.127.

### Comparison between *first-line chemotherapy* and surgery

As shown in Table 6, the 2 subgroups were well balanced in terms of clinical features. It is important to note that EPD was the preferred surgical approach in the neoadjuvant chemotherapy subgroup (probably due to disease progression) compared to the *surgery-first* subgroup (84% vs 50%: *P*-value <0.001). In those who received neoadjuvant chemotherapy, the regime comprised 2 (IQR: 2–3) cycles. For this reason, the interval from diagnosis to surgery in this group was 5.2 (SD: 4.5) months compared to 3.4 (SD: 2.8) months in the first-line surgery group (*P*-value < 0.001).

**Table 6:** Clinicopathological characteristics classified by *first-line chemotherapy* versus *surgery*

	First-line chemotherapy	Surgery first	*P*-value
	(n = 95)	(n = 34)	
Age, years (mean±SD) (median–percentiles)	67.60 (SD: 6.91)	65.55(SD: 10.26)	0.646
Gender			0.421
Male	81 (85.2%)	27 (79.4%)	
Female	14 (14.8%)	7 (2.0.6%)	
Smoking history			0.202
Never	40 (42.1%)	9 (26.5%)	
Former + current	55 (57.9%)	25 (63.5%)	
ECOG			0.295
0	49 (51.6%)	21 (63.8%)	
1 + 2	46 (48.4%)	13 (36.2%)	
Pre-op Hb			0.268
≤130	65 (68.4%)	20 (58.8%)	
>130	30 (31.6%)	14 (41.2%)	
Pre-op BMI			1.000
<25	31 (32.6%)	11 (32.3%)	
≥25	64 (67.4%)	23 (67.1%)	
Biopsy method			0.547
VATS	58 (60.1%)	21 (61.8%)	
Medical thoracoscopy	22 (23.7%)	6 (17.6%)	
Percutaneous CT/US guided	15 (16.2%)	7 (20.6%)	
Biopsy: histological analysis			0.729
Epithelioid	85 (89.5%)	32 (94.1%)	
Other	10 (1.0.5%)	2 (5.9%)	
Surgical procedure			< 0.001
PD	15 (15.8%)	17 (50%)	
EPD	80 (84.2%)	17 (50%)	
Interval: biopsy–surgery (months)	5.21 (SD: 4.50)	3.35 (SD: 2.83)	<0.001
Surgery: histological analysis			0.006
Epithelioid	65 (68.4%)	31 (88.2%)	
Non-epithelioid	30 (21.6%)	3 (11.8%)	
Sarcomatoid component			0.061
≤20%	73 (76.8%)	31 (91.2%)	
>20%	22 (23.2%)	3 (8.8%)	
Stage 8TNM			0.194
IA+IB	39 (41.1%)	19 (55.9%)	
II+III	56 (58.9%)	15 (44.1%)	
N Status			0.392
N0	44 (46.3%)	19 (55.9%)	
N+	51 (53.7%)	15 (44.1%)	

BMI: body mass index; CT/US: computed tomography/ultrasound; ECOG: Eastern Cooperative Oncology Group; EPD: extended pleurectomy/decortication; Hb: haemoglobin; N: node; PD: pleurectomy/decortication; pre-op: preoperative; SD: standard deviation; TNM: tumour/node/metastasis; VATS: video-assisted thoracoscopic surgery.

### Survival

**Figure 1:**
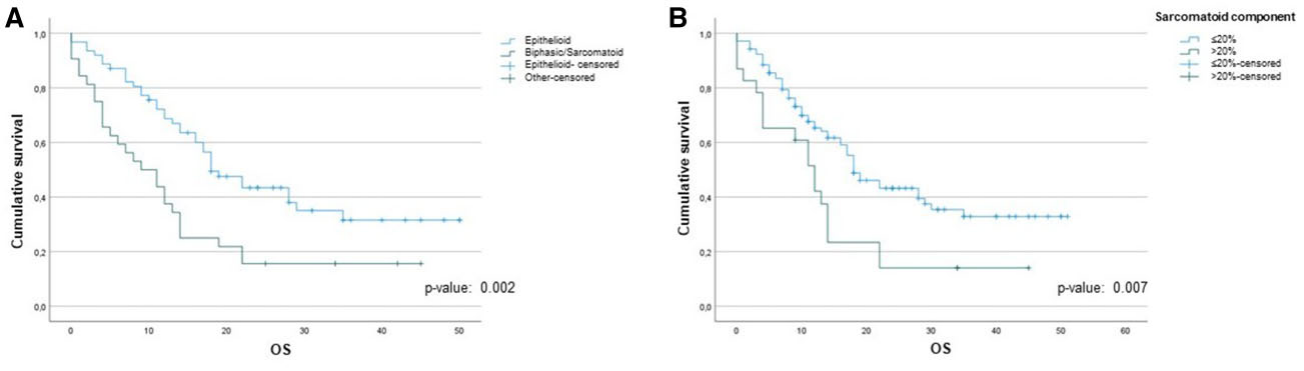
(**A**) Kaplan–Meier survival analysis of the malignant pleural mesothelioma (MPM) histopathological subtypes. (**B**) Kaplan–Meier survival analysis considering the percentage of post-resectional sarcomatoid component.

**Figure 2:**
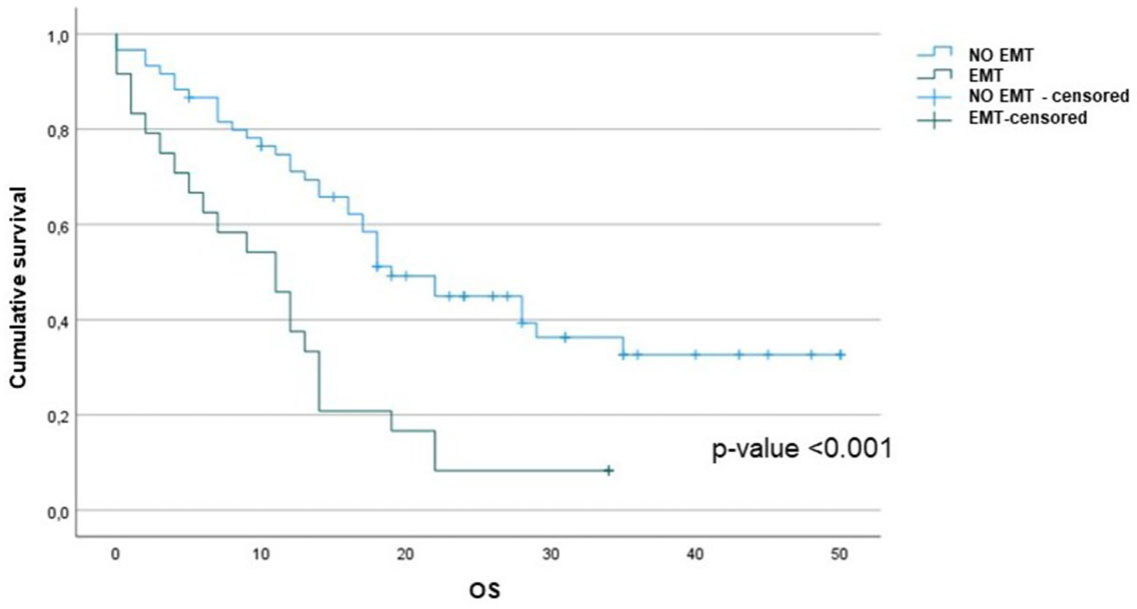
Kaplan–Meier survival analysis after neoadjuvant chemotherapy in patients who exhibited *positive vs negative discordance*.

**Figure 3:**
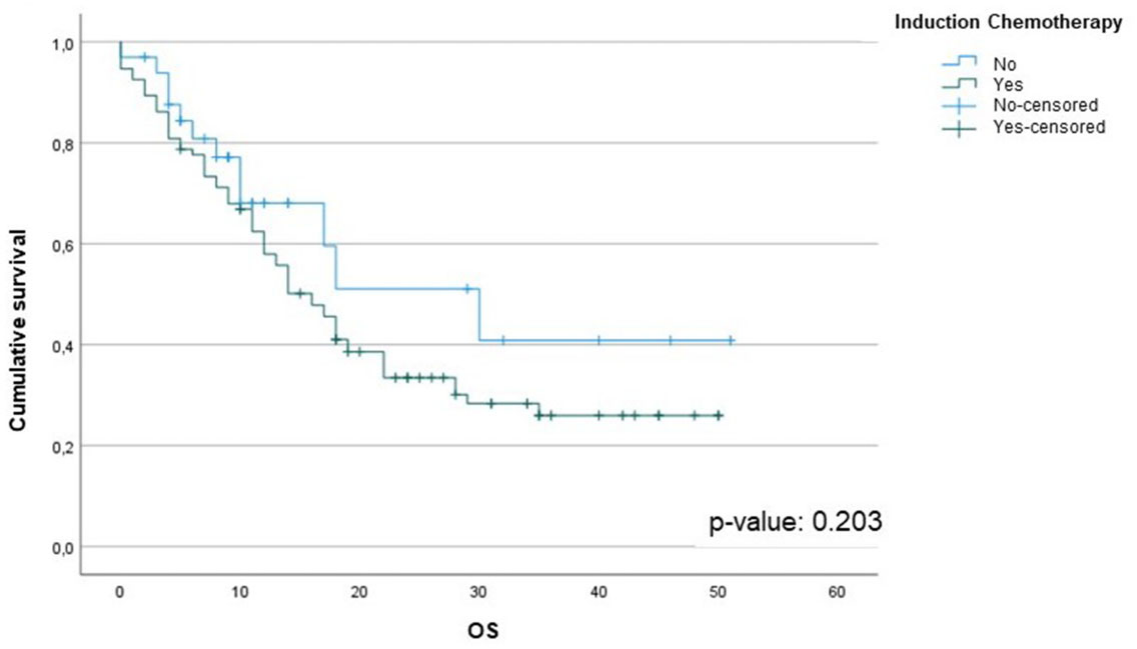
Kaplan–Meier survival analysis of patients who received neoadjuvant chemotherapy and those who had upfront surgery.

At the median follow-up of 17 months [IQR: 11.0–28.0] [Follow-up Index  = 1], 50 (39%) patients are still alive. We found that the OS from the date of diagnosis was associated with the histological subtype (epithelial vs non-epithelial) (*P*-value: 0.002) (Fig. 1A). Furthermore, OS was significantly reduced in those whose post-resectional histological sample contained more than 20% sarcomatoid component: 12 (95% CI: 8.7–15.3) months versus 18 (95% CI: 13.6–22.4) months, *P*-value: 0.007 (Fig. 1B). Overall survival was significantly reduced in those patients who received neoadjuvant chemotherapy and exhibited discordance compared to those who did not: 11 (95% CI: 6.2–15.8) months vs 19 (95% CI: 14.2 –23.8) months, *P*-value < 0.001 (Fig. 2). There was no difference in OS between those who received neoadjuvant chemotherapy and those who had upfront surgery: 16 (95% CI: 12.5–19.5) months versus 30 (95% CI: 11.6–48.4) months, *P*-value: 0.203 (Fig. 3). Cox univariate analyses identified that age, smoking status, preoperative haemoglobin levels, surgical procedure, tumour histological type, tumour thickness, sarcomatoid component, tumour stage, lymph node status and concordance/discordance were associated with OS (Table 7). After adjusting for these confounding factors, we found that the *concordant* subgroup showed a significantly better OS compared to patients who exhibited discordance between preoperative tissue diagnosis and post-resectional histological type (HR: 2.130; 95% CI: 1.210–3.747, *P*-value: 0.009; Fig. 5).

**Figure 4:**
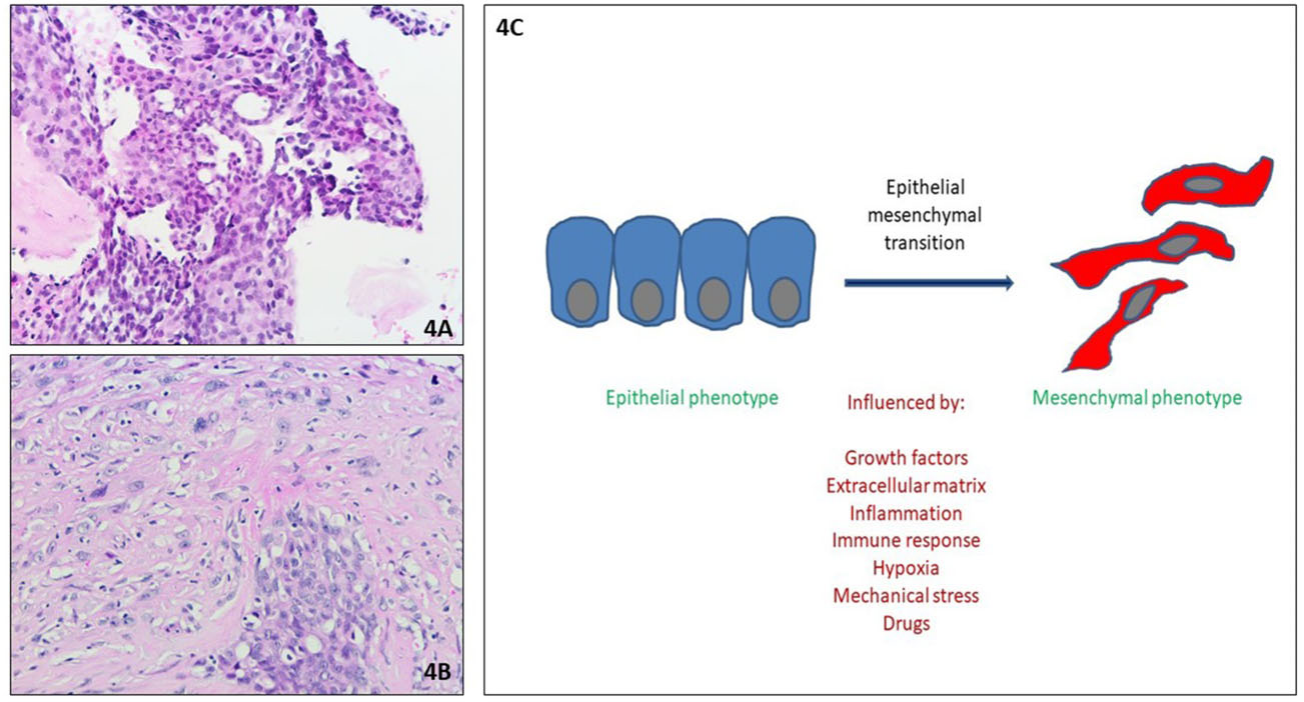
(**A**) Case of a patient with a diagnosis of epithelioid mesothelioma after a video-assisted thoracoscopic surgery pleural biopsy. (**B**) After 3 cycles of neoadjuvant chemotherapy, an extended pleurectomy/decortication was performed, and the final histopathological report showed a biphasic mesothelioma (sarcomatoid component > 80%. (**C**) Several factors can explain the epithelial-to-mesenchymal transition; one of these is the effect of drugs (including chemotherapy).

**Figure 5:**
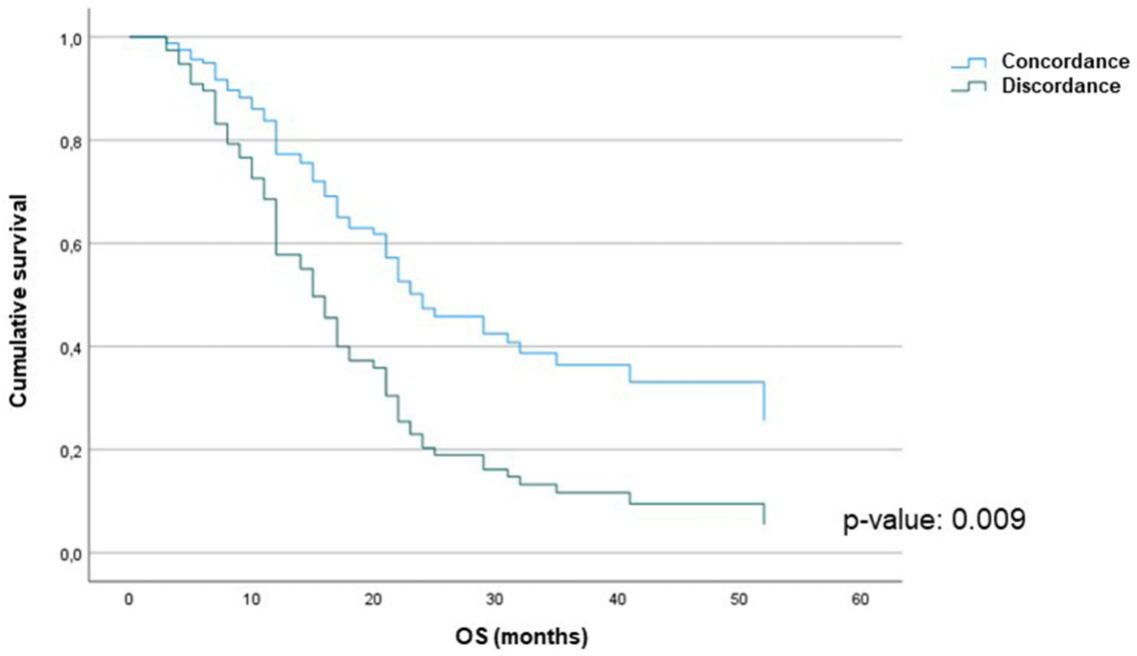
Kaplan–Meier survival analysis of *concordance versus discordance* subgroups after adjusting for predictive confounding factors.

**Table 7:** Univariate and multivariable Cox regression analysis of overall survival

Characteristics	Univariate Cox analysis			Multivariable Cox analysis		
	HR	95% CI	*P*-value	HR	95% CI	*P*-value
Age						
≤70	1	–	–	1	–	–
>70	1.565	1.201–2.459	0.032	1.838	1.113–3.036	0.017
Gender						
Male	1	–	–	–	–	–
Female	1.022	0.552–1.893	0.944	–	–	–
Smoking status						
Current+former	1			1	–	–
Never	0.710	1.033–2.700	0.036	1.701	1.020–2.839	0.042
ECOG						
0	1	–	–	–	–	–
1 + 2	1.670	0.742– 1.820	0.511	–	–	–
Pre-op HB						
≤130	1	–	–	1	–	–
>130	0.602	0.363– 0.998	0.049	0.619	0.349–1.097	0.100
Pre-op BMI						
<25	1	–	–			
≥25	0.832	0.520–1.332	0.445			
Biopsy method						
VATS	1	–	0.021	-	-	-
Percutaneous CT/US Guided	0.528	0.275–1.015	0.056	–	–	–
Medical Thoracoscopy	0.481	0.261–0.885	0.019	-	-	-
Biopsy: Histological diagnosis						
Epithelioid	1	–	–	–	–	–
Non-epithelioid	1.574	0.783–3.162	0.203	–	–	–
Surgical procedure						
PD	1	–	–	1	–	–
EPD	2.082	1.098–3.949	0.025	1.570	0.787–3.134	0.201
Surgery: histologial diagnosis						
Epithelioid	1	–	–	1	–	–
Non-epithelioid	2.313	1.449–3.693	<0.001	1.514	0.596–3.844	0.383
Sarcomatoid component						
≤20%	1	–	–	1	–	–
>20%	1.990	1.180–3.357	0.010	1.463	0.558–3.839	0.439
Tumour thickness	1.021	1.003–1.039	0.024	1.018	1.001–1.035	0.041
Stage						
IA+IB	1	–	–	1	–	–
II+III	1.623	1.011–2.603	0.045	0.423	0.093–1.926	0.266
N Status						
N0	1	–	–	1	–	–
N+	1.832	1.149–2.921	0.011	3.954	0.905–17.275	0.048
Concordance vs Discordance						
Concordance	1	–	–	1	–	–
Discordance	2.165	1.297–3.611	0.003	2.130	1.210–3.747	0.009
Induction chemotherapy						
No	1	–	–	1	–	–
Yes	1.481	0.797–2.752	0.214	–	–	–

BMI: body mass index; CI: confidence interval; CT/US: computed tomography/ultrasound; ECOG: Eastern Cooperative Oncology Group; EPD: extended pleurectomy/decortication; Hb: haemoglobin; HR: hazard ratio; N: node; PD: pleurectomy/decortication; pre-op: preoperative; VATS: video-assisted thoracoscopic surgery.

## DISCUSSION

We found cell type discordance between preoperative tissue diagnosis and post-resectional histological type in almost 1 in 3 patients undergoing neoadjuvant platinum/pemetrexed chemotherapy before pleurectomy/decortication. Those who demonstrated epithelial to non-epithelial cell type had significantly poorer survival than those who did not.

To explain these findings, we need to exclude some potential confounding factors. An obvious comment is that discordance in histological cell type between biopsy and resection is simply explained by the sampling error from small, isolated biopsy samples. However, we have found no association between discordance and the method of biopsy. Discordance was no greater following a needle biopsy than following surgical thoracoscopy. Indeed, there were significantly more cases diagnosed by the percutaneous method in the *concordant* group. Furthermore, there were cases in whom extensive, multi-site surgical biopsies demonstrated epithelial disease but at resection a biphasic tumour containing 80% sarcomatoid elements was found after chemotherapy. It is difficult to attribute such a result to a sampling error at biopsy. Importantly, there was also no difference in the maximum diameter or in the volume of biopsy material between those who demonstrated discordance and those who did not.

If one assumes that the non-epithelial cell type predominates in the latter phases of the natural history of mesothelioma, then the following ideas need to be considered. First, there was no evidence that discordance was seen in a more advanced stage of the disease, as demonstrated by similar maximum tumour thickness and pathological nodal positivity.

There may have been a longer time delay in those who developed discordance, which may be explained by the higher proportion who underwent neoadjuvant chemotherapy and therefore were operated on at a median duration of around 8 weeks later. It is possible that in this relatively short interval there may have been natural tumour progression towards the non-epithelial cell type in the context of chemoresistance.

Because we cannot confidently explain our findings by invoking any of the above clinical confounding factors, we can hypothesize about molecular mechanisms that may explain our results. If one assumes that all mesotheliomas contain populations of both cell types, then if chemotherapy preferentially acts on the epithelial component of the disease, it may allow proliferation of the sarcomatoid component. This situation would result in an increased proportion of biphasic disease in those who received chemotherapy.

A further and more robust explanation is that platinum chemotherapy induces the phenomenon of EMT at the molecular level, thus increasing the proportion of the sarcomatoid cell-type. Epithelial-to-mesenchymal transition is characterized by an upregulation of mesenchymal markers or some miRNAs or non-coding RNAs and results in increased tumour cell mobility, invasiveness, apoptosis resistance and even resistance to chemotherapy [9]. Epithelial-to-mesenchymal transition may be induced by transforming growth factor β, the main inducer of EMT, and by oxidative stress (Fig. 4).

There is animal model evidence to support our hypothesis. Chemotherapy itself has been reported to induce irreversible EMT through endoplasmic reticulum stress. Lung adenocarcinoma cell lines treated with common chemotherapeutic agents including pemetrexed, vinorelbine and gemcitabine demonstrated EMT as revealed by an increase in the expression of mesenchymal markers [10].

Previous series have reported similar levels of histological discordance of around 20% [5, 11], but these series involved open pleural biopsy and resection predominantly by extrapleural pneumonectomy [4]. Similarly, both of these series reported that neoadjuvant chemotherapy from an epithelial biopsy to a non-epithelial resected specimen was the most frequent finding. Bueno *et al.* found that 44% of those with a final diagnosis of non-epithelial cells were initially misdiagnosed with the epithelial subtype [11]. In their series of 95 patients with a final diagnosis of MPM, Greillier *et al.* [4] found that, of the 87 patients classified as the epithelial type after the initial thoracoscopy, 12 (13.8%) were found subsequently to be of the biphasic subtype at the final diagnosis. Unlike our study, these series did not consider the influence of neoadjuvant chemotherapy on the subsequent finding of histological discordance.

Although we found no difference in the amount of biopsy material, we did not record the number of sites from which preoperative material was obtained; however, discordance has been associated with a lower number of biopsy samples. Chirieac [5] reported that the median number in the concordant group was still only 3 whereas in those with discordant histological types, the mean number of tissue blocks in the initial biopsies was significantly lower. Similarly, the number of tissue blocks examined was higher in patients with concordant histological types than in those with discordant histological types for thoracoscopic biopsies. Indeed, in a multivariable analysis, OS was independently predicted by the histological type in the resection specimen but not that in the biopsy.

An argument in favour of a sampling error as a reason for histological discordance is the understanding of intra-tumoural heterogeneity [12]. The suggestion that mesothelioma tumour cells exhibit polyclonal dissemination and evolve separately at different locations within the thorax would explain the varying geographical proportion of epithelioid and sarcomatoid phenotypes. Thus, isolated biopsies, even multiple ones, may not truly represent the tumour architecture.

It is important to understand that no individual mesothelioma tumour contains only 1 specific histological cell type or even belongs to a subdivision based on molecular features [13]. Rather, the molecular profile and the prognosis of MPM appear to be better explained by a model containing inherent continuity of the tumour phenotypes typified by strong differences in the expression of proangiogenic and immune checkpoint genes [14]. Although we did not find any clear relationship between the method of diagnostic biopsy and the subsequent finding of EMT, we had only limited data on the comparative volumes of diagnostic tissue. Similarly, we have diagnostic biopsies from many hospitals, and we cannot guarantee quality control because we have not had an independent review of the histological data. The biopsy reports were issued by a large number of potentially less experienced pathologists, whereas the resection specimens were reported by a small number of pathologists who regularly review a large number of specimens. Having said that, the pathologists involved were all consultants. Also, all tissue biopsies of insufficient size were reported. Based on the histopathology report, all biopsies satisfied the basic requirements.

Another limitation of this study is that the selection algorithm for neoadjuvant treatment was dependent on the decision of the referral center, which could lead to selection bias. This assumption is corroborated by the higher rate of EPD in the first-line chemotherapy group. The objective selection criteria were determined from the CT scan and, within our unit, outside the clinical trial pathway, upfront surgery was offered to cT1/T2N0M0 patients.

Future work should be directed at more detailed comparative genomic analysis of those tumours that do and do not exhibit EMT. If it were possible to identify patients at greatest risk, then the use of platinum-based treatment may be avoided.

Our findings may have several implications. In clinical practice, an adequate diagnostic pleural biopsy should be as extensive as possible with samples from at least 3 separate areas of interest identified on presurgical imaging. For example, there has been a call for an international recommendation of the number and sites of diagnostic pleural biopsies [6]. Interestingly, we found no evidence to support performing a pleural biopsy with the patient under general anaesthesia because discordance was not reduced by this approach. We do suggest that a re-biopsy should be considered after neoadjuvant chemotherapy, if we assume that EMT transition should preclude surgery because of the relatively poor outcome compared to a second-line systemic therapy or even in a patient who would benefit from adjuvant immunotherapy.

There has been support for the use of neoadjuvant chemotherapy in otherwise resectable disease [15]. The assumption has been that this strategy may be beneficial because (1) the patient is more likely to be compliant with a complete course of chemotherapy if it is given before major surgery or (2) there may be a favourable response if tumour volume is reduced, thereby facilitating macroscopic clearance. This period may also act as a form of “biological selection” such that surgery is not then offered in progressive, chemoresistant cases. Although our results may support the latter hypothesis, the subsequent improvement in surgical outcome from selecting out those whose tumours become non-epithelial after chemotherapy may be offset by an overall reduction in survival of those with potentially resectable tumours.

The potential for inducing EMT from platinum-based chemotherapy should be borne in mind when appraising clinical trials on mesothelioma therapy. The results of the soon-to-report MARS2 trial [16] of platinum/pemetrexed chemotherapy with or without pleurectomy/decortication must be interpreted carefully. Although the randomization could reasonably be assumed to have resulted in equal EMT in both groups, it will not be valid to perform subgroup analysis of surgery versus no surgery on the basis of diagnostic cell type in 1 group and of postresection in the other group. More controversially, if neoadjuvant chemotherapy may prove to be detrimental because of EMT, then can one conclusively rule out surgical treatment based on this trial? The results of the EORTC 1205 trial [17] of neoadjuvant versus adjuvant platinum/pemetrexed before or after pleurectomy/decortication will go some way to answer these doubts.

In non-surgical trials, this high rate of histological discordance implies that prognostic statements based on the subgroup analysis of cell type may be misleading. Furthermore, in trials of second-line systemic therapies after platinum-based regimes, a further biopsy to confirm histological cell type would be advisable to stratify response rates.

## CONCLUSION

For the benefit of our patients, clinical assessment before radical surgery should be maximized to improve postoperative survival. Detailed, multiple, multi-site pleural biopsies are needed to reduce cell type inaccuracy, and re-biopsy after chemotherapy should be considered to exclude transition to biphasic disease. Considering that our results did not show any survival difference between upfront surgery and neoadjuvant chemotherapy, the assumed gold standard of preoperative chemotherapy should be challenged, and upfront pleurectomy/decortication should be considered for early-stage (T1/T2N0M0) resectable epithelial disease pending the results of the previously mentioned randomized trials. However, EMT-induced cell-type discordance should be noted as a potential confounding factor in the interpretation of survival in these and other non-surgical studies.

## Data Availability

The data underlying this article will be shared on reasonable request to the corresponding author.

## Notes

Presented at the International Association for the Study of Lung Cancer 2022 World Conference on Lung Cancer, Vienna, Austria, 6–9 August 2022.

## Abbreviations

CT: Computed tomography
EMT: Epithelial-to-mesenchymal transition
EPD: Extended pleurectomy/decortication
IQR: Interquartile ranges
MPM: Malignant pleural mesothelioma
N1: Resectable nodal disease
OS: Overall survival
PD: Pleurectomy/decortication
SD: Standard deviation
US: Ultrasound
